# Neural networks associated with body composition in frontotemporal dementia

**DOI:** 10.1002/acn3.50869

**Published:** 2019-08-28

**Authors:** Rebekah M. Ahmed, Ramon Landin‐Romero, Cheng T. Liang, Julia M. Keogh, Elana Henning, Cherie Strikwerda‐Brown, Emma M. Devenney, John R. Hodges, Matthew C. Kiernan, I. Sadaf Farooqi, Olivier Piguet

**Affiliations:** ^1^ Memory and Cognition Clinic, Department of Clinical Neurosciences Royal Prince Alfred Hospital Sydney Australia; ^2^ Central Sydney Medical School and Brain & Mind Centre The University of Sydney Sydney Australia; ^3^ School of Psychology and Brain & Mind Centre The University of Sydney Sydney Australia; ^4^ ARC Centre of Excellence of Cognition and its Disorders Sydney Australia; ^5^ University of Cambridge Metabolic Research Laboratories and NIHR Cambridge Biomedical Research Centre, Wellcome Trust‐MRC Institute of Metabolic Science, Addenbrooke’s Hospital Cambridge UK

## Abstract

**Background:**

Frontotemporal dementia (FTD) is associated with complex changes in eating behavior and metabolism, which potentially affect disease pathogenesis and survival. It is currently not known if body composition changes and changes in fat deposition also exist in FTD, the relationship of these changes in eating behavior and appetite, and whether these changes are centrally mediated.

**Methods:**

Body composition was measured in 28 people with behavioral‐variant frontotemporal dementia (bvFTD), 16 with Alzheimer’s disease (AD), and 19 healthy controls, using dual energy x‐ray absorptiometry. Changes in body composition were correlated to brain grey matter atrophy using voxel‐based morphometry on high‐resolution magnetic resonance imaging.

**Results:**

Behavioral‐variant FTD was characterized by changes in body composition, with increased total fat mass, visceral adipose tissue area (VAT area), and android: gynoid ratio compared to control and AD participants (all *P* values < 0.05). Changes in body composition correlated to abnormal eating behavior and behavioral change (*P *< 0.01) and functional decline (*P *< 0.01). Changes in body composition also correlated to grey matter atrophy involving a distributed neural network that included the hippocampus, amygdala, nucleus accumbens, insula, cingulate, and cerebellum – structures known to be central to autonomic control – as well as the thalamus, putamen, accumbens, and caudate, which are involved in reward processing.

**Conclusions:**

Changes in body composition and fat deposition extend the clinical phenomenology in bvFTD beyond cognition and behavior, with changes associated with changes in reward and autonomic processing suggesting that these deficits may be central in FTD

## Introduction

The effects of neurodegenerative dementia syndromes extend beyond cognition and behavior to involve the body’s key physiological systems including eating and metabolism, and the autonomic nervous system.^1^ Changes in eating behavior are well documented in frontotemporal dementia (FTD),^2^, ^3^, ^4^ arising from complex interactions between structures controlling cognitive‐reward, autonomic, and neuroendocrine modulation of eating behavior.^3^

The metabolic changes found in FTD reflect complex disturbances that go beyond those expected from an increased oral intake. Indeed, patients show a smaller increase in body mass index (BMI) than expected for oral intake and are found to be hypermetabolic, with increased resting energy expenditure.^5^ These changes, which are present across the FTD and amyotrophic lateral sclerosis (ALS) spectrum, may influence disease progression, with increasing eating behavior associated with an improved survival.^6^ The mechanisms underlying this phenomenon are not fully understood, but may be related to changes in lipid levels, with lipid levels along the ALS‐FTD spectrum related to fat intake, and increased cholesterol levels improving survival.^6^ .Anecdotally, ALS patients also complain of changes in abdominal girth with visceral fat deposition potentially modifying survival 7

It is currently not known if changes in body composition including fat distribution also occur in FTD, and whether these changes are related to changes in eating behavior, or may represent a complex interaction between eating behaviour and the neurodegenerative process with central changes in the neural structures controlling reward and autonomic function also potentially playing a role.

The current study aimed to determine changes in body composition and fat deposition in patients diagnosed with behavioral‐variant FTD (bvFTD) using dual energy x‐ray absorptiometry (DEXA) scans.^8^ Targeted key measures of body composition included total lean mass, fat mass, android: gynoid ratio (a measure of the amount of fat deposited around the trunk), which has previously been correlated to insulin resistance and lipid levels,^9^ and visceral adipose tissue area: VAT area (a measure of visceral adipose tissue deposition). The relationship between these parameters, behavioral change, and neural networks was examined to determine if peripheral body composition changes are associated with centrally mediated processes by the neurodegenerative process, particularly in relation to reward and autonomic processing.

## Methods

### Participants

Forty‐four patients with dementia (28 bvFTD, 16 AD) were recruited from Frontier, the FTD research clinic based at the Brain and Mind Centre, University of Sydney, Australia. These individuals were compared with 19 age‐ and sex‐matched healthy controls. All patients underwent neurological review, cognitive assessment, and met current clinical diagnostic criteria for probable bvFTD or Alzheimer’s disease (AD).^10^, ^11^, ^12^, ^13^ Disease severity was established using the Frontal Rating Scale (FRS, lower score indicates worse function),^14^ and disease duration by estimated onset to date of assessment. General cognitive functioning was established with the Addenbrooke’s Cognitive Examination‐III (ACE‐III).^15^ The presence of abnormalities in the *C9orf72, TDP43, FUS, GRN,* and *MAPT* genes was examined in all FTD patients. Healthy controls were recruited from a volunteer database, scored above 88/100 on the ACE‐III, the control group was matched to the bvFTD group for BMI.

### Ethics

This study was approved by the South Eastern Sydney Local Health District and the University of New South Wales human ethics committees. Written informed consent was obtained from each participant and/or their primary caregiver.

### DEXA scans and whole body composition

Participants completed a single whole‐body scan on a Hologic Horizon A (SN‐300616M) (Hologic Inc., Bedford, MA www.hologic.com). The Hologic whole‐body scanning dimension was 196 cm by 68 cm, which ensured adequate separation of the arms from the trunk in the whole‐body positioning.

Dual energy x‐ray absorptiometry body scan data were analyzed with the QDR system software for Windows (XP) Hologic software APEX 5.6.0.5 (Hologic). Total mass (grams), lean mass (grams), fat mass (grams), and fat percent were calculated for the whole body and for individual regions of interest: the head, upper limbs, lower limbs, and trunk. Regions of interest were defined as follows: head = immediately below the mandible; trunk = enclosing the chest, midriff, and pelvis; the left and right upper limbs = medial to the head of the humerus; and left and right legs = boundary placed outside of the thigh through to the middle of both legs through the femoral neck and lateral to the pubic ramus. Visceral abdominal fat (visceral adipose tissue area:VAT area in cm^2^) was initially identified automatically followed by manual adjustment to ensure that the lateral edges of the VAT area were correctly defined. From the DEXA scan, key measures of total lean mass, total fat mass, percentile fat (age matched):amount of fat matched to a population of similar age, android: gynoid ratio: fat in android (trunk area) compared to gynoid area,  and visceral adipose tissue area (VAT area): fat deposited around visceral organs were obtained for each participant.

### Behavioral measurements

In addition to measurements of body composition and BMI, changes in eating behavior were measured using caregiver‐based questionnaires: the Appetite and Eating Habits Questionnaire (APEHQ)^3^ and the Cambridge Behavioural Inventory (CBI).^16^ These surveys were completed on the same day the DEXA scan was obtained and were felt to be representative of eating behavior and general behavior over the preceding 6 months.^3^ Height and weight were measured barefoot and BMI was calculated (weight in kilograms/height in meters squared).

### Neuroimaging

#### Magnetic resonance imaging acquisition and preprocessing

Participants underwent whole‐brain magnetic resonance imaging (MRI) in a 3‐Tesla scanner (completed within 14 days on average to the DEXA scan). High‐resolution T1 images were obtained using the following protocol: 256 × 256, 200 slices, 1‐mm^2^ in‐plane resolution, 1‐mm slice thickness, echo time/repetition time = 2.6/5.8 ms, flip angle = 8°. Brain scans were available for 14 AD patients, 26 bvFTD patients, and 19 healthy controls. MRI data were analyzed with FSL‐VBM, a voxel‐based morphometry analysis^17^ using the FSL‐VBM toolbox from the FMRIB software package (http://www.fmrib.ox.ac.uk/fsl/fslvbm/index.html)^18^ (see Ref. 19, 20 for full details of the methods).

#### VBM analyses

Voxel‐wise general linear models were applied to identify differences in grey matter intensity through permutation‐based nonparametric testing^21^ with 5000 permutations per contrast. Pairwise differences in cortical grey matter density between groups (AD, bvFTD, and controls) were assessed using t‐tests. Clusters were extracted using a voxel‐wise method, uncorrected for multiple comparisons, at *P* < 0.005.

Next, correlations between BMI, android: gynoid ratio, and VAT area, and regions of grey matter atrophy were investigated in each patient group separately (i.e., bvFTD and AD, given the differences in behavior). For additional statistical power, each patient group was analyzed with the control group and a covariate‐only statistical model with a [−1] t‐contrast was used, providing an index of association between grey matter atrophy and increases in the behavioral measure. Statistical significance was set at *P* < 0.005, uncorrected for multiple comparisons, with a conservative cluster extent threshold of 100 voxels. This approach is designed to minimize Type I error while balancing the risk of Type II error.

Anatomical locations of significant results were overlaid on the MNI standard brain using the mricron software (https://www.nitrc.org/projects/mricron), with maximum voxel coordinates provided in the stereotaxic space. Anatomic labels were determined with reference to the Harvard‐Oxford probabilistic cortical and subcortical atlases.

### Statistical analyses

#### Demographic and physiological variables

Analyses were conducted using IBM SPSS statistics (version 24.0). Kolmogorov–Smirnov tests were run to determine suitability of variables for parametric analyses. One‐way analyses of variance (ANOVA), followed by Tukey post hoc tests, were used to determine group differences in demographic and clinical variables. Categorical variables were analyzed using Chi‐squared analyses. Independent t‐tests were used to determine differences between bvFTD and AD for disease duration, abnormal behavior (total CBI, CBI behavioral), and eating behavior (APEHQ, CBI eating) (*P* ≤ 0.05 regarded as significant). Measurements of body composition (BMI, total lean mass, fat mass, percentile fat mass age matched, android: gynoid ratio, and VAT area) were also explored using ANOVA, followed by Tukey post hoc tests (*P* < 0.05 regarded as significant). The relationship between changes in body composition, disease duration, BMI, eating behavior (APEHQ total score) cognitive status (ACE‐III), and behavioral measures (CBI total, eating subscores) was further explored using Pearson rank correlations corrected for multiple comparisons (*P* ≤ 0.01 regarded as significant).

## Results

Participant groups were matched for sex distribution, age, and disease duration (Table 1, all *P* values > 0.132). Group differences were observed on measures of cognition (ACE‐III), behavioral measures, and eating behavior (Table 1) and were in keeping with the known behavior of the diagnostic groups. In the bvFTD group, seven patients were found to have the *C9orf72* expansion.

**Table 1:** Demographics and clinical characteristics in bvFTD, AD, and healthy controls.

	bvFTD (n = 28)	AD (n = 16)	HC (n = 19)	*F* value	*P* value	Post hoc
Sex (F:M)	5:23	6:10	6:13	2.2^1^	ns	–	
Age (years)	60.9 ± 7.0	60.3 ± 6.07	62.9 ± 6.9	0.794	ns	–	
BMI (kg/m^2^)	30.3 ± 4.9	24.5 ± 3.4	27.2 ± 2.8	8.8	*P* < 0.001	bvFTD>AD	
ACE‐III Total (max 100)	74.7 ± 16.1	62.3 ± 15.9	94.8 ± 2.9	27.2	*P* < 0.001	HC>patients; bvFTD>AD	
Disease duration (years)	6.7 ± 4.7	4.4 ± 2.2	–	1.5^2^	ns	–	
FRS Rasch score^3^	−0.98 ± 1.4	0.101 ± 1.1	–	2.1^2^	*P <* 0.05	bvFTD<AD	
APEHQ Total	58.8 ± 45.7	21.0 ± 19.1	–	2.8^2^	*P* < 0.01	bvFTD>AD	
CBI Total	72.9 ± 10.8	45.1 ± 23.0	–	2.9^2^	*P* < 0.01	bvFTD>AD	
CBI eating Total	6.9 ± 5.2	2.9 ± 2.5	–	2.8^2^	*P* < 0.01	bvFTD>AD	

Values are expressed as mean ± standard deviation. AD, Alzheimer’s disease; ACE‐III, Addenbrooke’s Cognitive Examination‐III; APEHQ, Appetite and Eating Habits Questionnaire; BMI, body mass index; bvFTD, behavioral‐variant frontotemporal dementia; CBI, Cambridge Behavioural Inventory; F, value from ANOVA; FRS, Frontotemporal dementia Rating Scale; HC, healthy controls; ns, not significant.

1 Chi‐squared test.

2 *t*‐value.

3 The FRS provides logit scores ranging from 4.12 (very mild) to −4.99 (very severe).

### Eating behavior

Based on caregiver surveys, bvFTD showed more severe eating disturbance than AD on the APEHQ (*t* = 2.8, *P* = 0.005), CBI total (*t* = 2.9, *P* = 0.005), and CBI eating (*t* = 2.8, *P* = 0.005) scores.

### BMI and DEXA scan results

**Figure 1:**
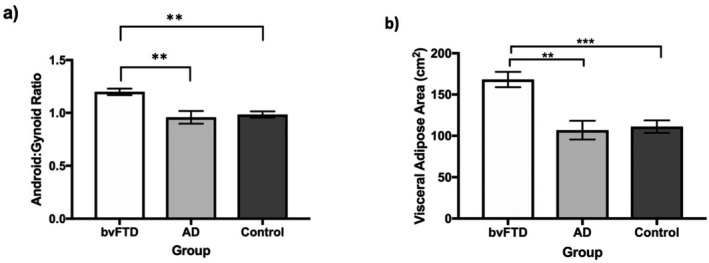
Android: gynoid ratio and VAT area in patient groups. **P < 0.01 *** *P* < 0.001.

The bvFTD group had a higher BMI than the AD (*P* = 0.001), but the difference in BMI between the control and bvFTD groups was not statistically significant. Measures of body composition showed the bvFTD group to have an increased total fat and lean mass and percentile fat age matched compared to both the control and AD groups (all *P* values < 0.05) (Table 2, in Fig. 1). The bvFTD group also exhibited an increased android: gynoid ratio compared with the AD (*P *= 0.003) and control (*P *= 0.006) groups. Finally, the bvFTD group showed an increased VAT area compared to both the AD (*P *= 0.001) and control groups (*P *= 0.001), despite being matched to the control group for BMI.

**Table 2:** DEXA scan results.

	bvFTD (n = 28)	AD (n = 16)	HC (n =19)	*F* value	*P* value
BMI	30.3 ± 4.9	24.5 ± 3.4	27.2 ± 2.8	8.8	0.001 AD<bvFTD
Total Lean Mass (g)	63282 ± 12311	50713 ± 12228	54369 ± 11693	6.3	0.003 AD<bvFTD, C<bvFTD
Total Fat mass	26149 ± 7509	19224 ± 7880	21590 ± 4806	5.7	0.000 AD<bvFTD, C<bvFTD
Percentile fat age matched %	50.1 ± 25.9	28.2 ± 25.4	31.9 ± 21.1	5.2	0.008 AD<bvFTD, C<bvFTD
Android: gynoid ratio	1.2 ± .17	.96 ± .23	.98 ± .14	8.1	0.009 AD<bvFTD, C<bvFTD
Visceral adipose tissue area (VAT area) cm^2^	163.2 ± 49.0	106.9 ± 49.7	111.2 ± 32.9	10.9	0.000 AD<bvFTD, C<bvFTD

Values are expressed as mean ± standard deviation. AD, Alzheimer’s disease; bvFTD, behavioral‐variant frontotemporal dementia; F, value from ANOVA; HC, healthy controls.

### Correlations of DEXA scan indices with behavioral measures

When all groups were combined due to small sample sizes, android: gynoid ratio was positively correlated with behavioral and eating changes as reflected by the CBI total (*r* = 0.307, *P* = 0.01), CBI behavioral (*r* = 0.301, *P* = 0.01), total CBI eating score (*r* = 0.305, *P* = 0.01), and BMI (*r* = 0.348, *P* = 0.006). VAT area was positively correlated with CBI total (*r* = 0.356, *P* = 0.004), CBI behavioral (*r* = 0.423, *P* = 0.004), CBI eating (*r* = 0.355, *P* = .004), and negatively correlated with the FRS (*r* = −0.350, *P* = 0.001). Finally, BMI was positively correlated with CBI eating (*r* = 0.300, *P* = 0.01) and CBI behavioral (*r* = 0.353, *P* = 0.005), and negatively correlated with the FRS (*r* = −0.350, *P* = 0.003).

### Neuroimaging results

#### Brain atrophy analyses

Patterns of brain atrophy were consistent with those typically found in these patient groups (Table S1 and Figure S1).

#### Imaging correlations (Table 3 and Figs. 2‐4)

**Figure 2:**
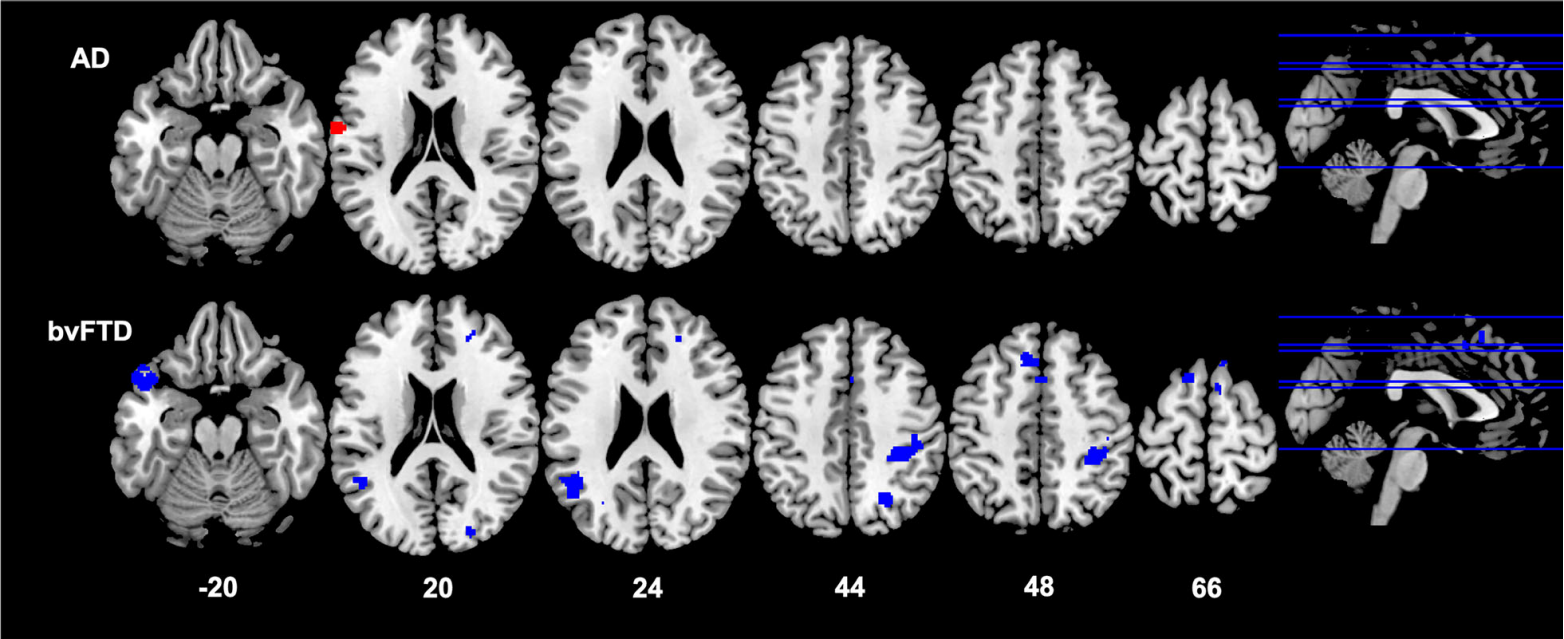
Neural correlates of BMI. Results from voxel‐based morphometry analyses illustrating correlations between an increase in BMI and decrease in grey matter density in AD (red) and bvFTD (blue) patients. All analyses are reported at *P* < 0.005 voxel‐wise, uncorrected for multiple comparisons with minimum cluster size of 100 voxels. The left side of the image is the left side of the brain. Numbers below each slice refer to MNI z‐coordinates. AD, Alzheimer’s disease; BMI, body mass index; bvFTD, behavioral‐variant frontotemporal dementia

**Figure 3:**
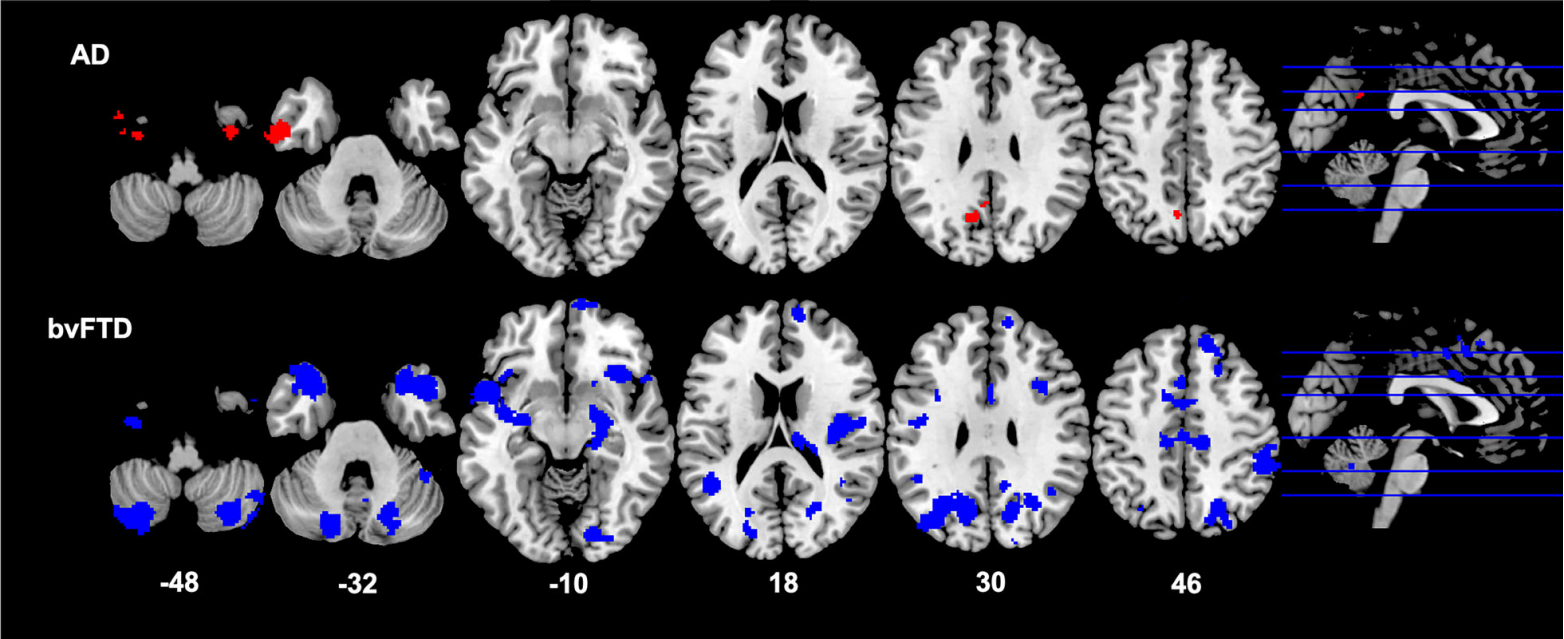
Neural correlates of VAT Area. Results from voxel‐based morphometry analyses illustrating correlations between an increase in VAT area and decrease in grey matter density in AD (red) and bvFTD (blue) patients. All analyses are reported at *P* < 0.005 voxel‐wise, uncorrected for multiple comparisons with minimum cluster size of 100 voxels. The left side of the image is the left side of the brain. Numbers below each slice refer to MNI z‐coordinates. AD, Alzheimer’s disease; bvFTD, behavioral‐variant frontotemporal dementia

**Figure 4:**
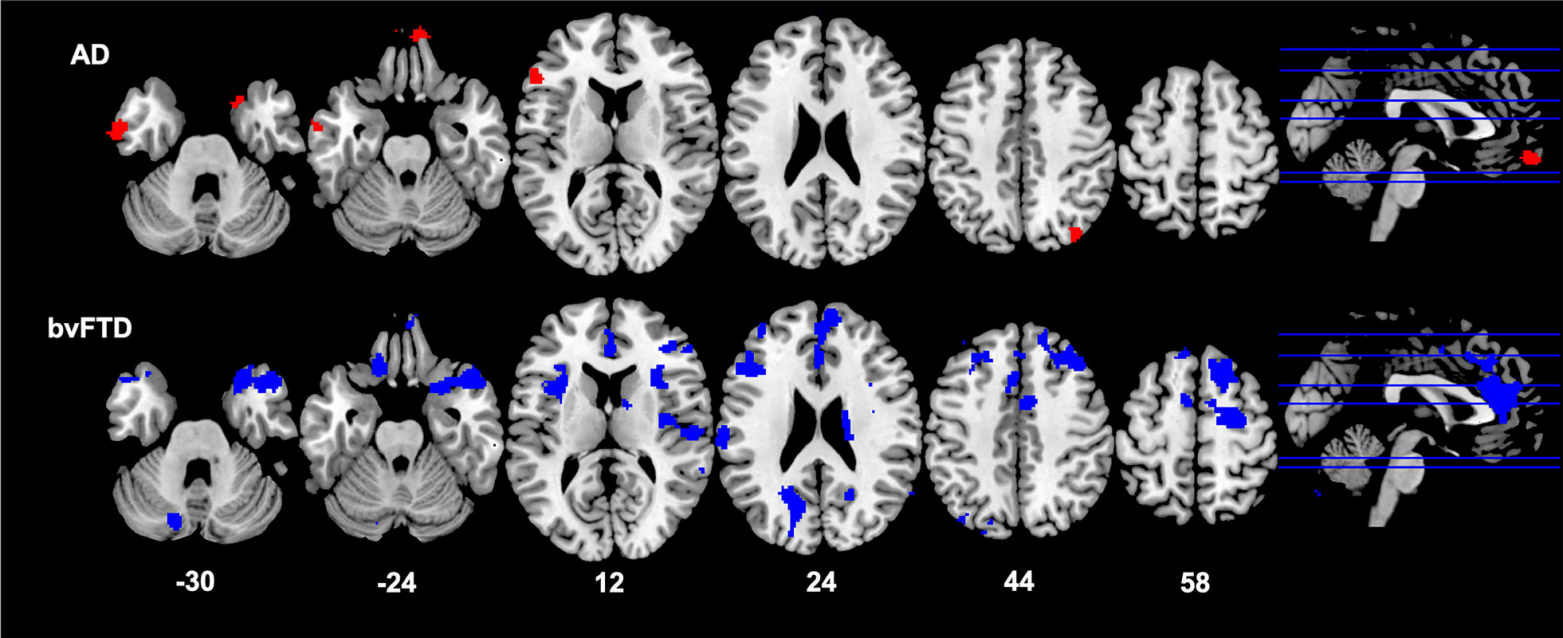
Neural correlates of android: gynoid ratio. Results from voxel‐based morphometry analyses illustrating correlations between an increase in android: gynoid ratio and decrease in grey matter density in AD (red) and bvFTD (blue) patients. All analyses are reported at *P* < 0.005 voxel‐wise, uncorrected for multiple comparisons with minimum cluster size of 100 voxels. The left side of the image is the left side of the brain. Numbers below each slice refer to MNI z‐coordinates. AD, Alzheimer’s disease; bvFTD, behavioral‐variant frontotemporal dementia

In the bvFTD group, higher BMI was associated with lower grey matter volume in the left superior frontal gyrus, temporal pole, insula, anterior cingulate cortex, and superior parietal region (Fig. 2). In this group, increasing VAT volume (Fig. 3) correlated with decreasing grey matter volume in a distributed bilateral network including the temporal pole, insula, accumbens, caudate, putamen, amygdala, and thalamus. These key areas extended anteriorly to prefrontal cortices and anterior cingulate cortex, and posteriorly to include parietal regions, lingual gyrus, occipital cortex, as well as the cerebellum. Increasing android: gynoid ratio (Fig. 4) correlated with decreased grey matter volume in a similar network involving the bilateral frontal pole, right temporal pole, insula, putamen, caudate, thalamus, left parietal structures including the cuneus and precuneus, and the cerebellum (Table 3).

**Table 3:** Voxel‐based morphometry results showing regions of significant grey matter intensity decrease that covary with increase in BMI, increase in VAT area, and increase in Android: Gynoid ratio in bvFTD combined with Controls and AD combined with Controls.

	Regions	Side	Number of voxels	MNI coordinates		
				*x*	*y*	*z*
Regions that covary with BMI increase						
bvFTD and Controls	Superior frontal gyrus, Paracingulate gyrus	L	508	−10	28	48
	Angular gyrus, Supramarginal gyrus, Lateral occipital cortex, Middle temporal gyrus	L	495	−44	−52	24
	Temporal pole, Orbitofrontal cortex, Insula	L	307	−48	10	−20
	Postcentral gyrus, Superior parietal lobule, Supramarginal gyrus	R	287	34	−36	44
	Superior frontal gyrus, Paracingulate gyrus, Anterior cingulate cortex	R	162	10	26	66
AD and Controls	Postcentral gyrus, Precentral gyrus, Central opercular cortex	L	121	−66	−4	20
Regions that covary with VAT increase						
bvFTD and Controls	Temporal pole, Orbitofrontal cortex, Insula, Caudate, Putamen, Accumbens, Pallidum, Parahippocampal gyrus, Hippocampus, Amygdala, Thalamus	R	2827	48	16	−34
	Temporal pole, Orbitofrontal cortex, Medial prefrontal cortex, Frontal pole, Insula, Central opercular cortex, Parahippocampal gyrus, Amygdala, Putamen, Pallidum	L	2434	−28	24	−36
	Frontal pole, Medial prefrontal cortex, Superior frontal gyrus, Supplementary motor cortex, Paracingulate gyrus	R	1701	10	68	−10
	Cerebellum	R	1459	46	−52	−56
	Lateral occipital cortex, Precuneus, Posterior cingulate cortex, Angular gyrus, Cuneus, Occipital pole	L	1406	−18	−84	18
	Lateral occipital cortex, Precuneus, Posterior cingulate cortex, Angular gyrus, Cuneus, Occipital pole	R	989	24	−72	16
	Cerebellum	L	934	−30	−74	−60
	Supplementary motor cortex, Anterior cingulate cortex, Superior frontal gyrus, Paracingulate gyrus	L & R	686	−2	2	46
	Middle temporal gyrus, Lateral occipital cortex, Angular gyrus, Supramarginal gyrus	L	527	−46	−58	4
	Planum temporale, Heschl’s gyrus, Superior temporal gyrus, Central opercular cortex, Parietal operculum, Postcentral gyrus, Insula	R	514	60	−12	4
	Supramarginal gyrus, Angular gyrus, Postcentral gyrus	R	368	52	−42	48
	Precentral gyrus, Middle frontal gyrus, Inferior frontal gyrus	R	280	38	−2	38
	Precentral gyrus, Posterior cingulate cortex, Supplementary motor cortex	L & R	203	14	−24	48
	Lingual gyrus, Fusiform gyrus, Lateral occipital cortex, Intracalcarine cortex	L	187	−18	−84	−2
	Cerebellum	R	176	46	−46	−32
	Postcentral gyrus, Precentral gyrus, Middle frontal gyrus, Inferior frontal gyrus	L	150	−40	−8	30
	Fusiform cortex, Inferior temporal gyrus	L	136	−36	−10	−48
	Angular gyrus, Supramarginal gyrus, Lateral occipital cortex	R	128	40	−54	24
	Lateral occipital cortex, Middle temporal gyrus	R	120	44	−60	2
	Cerebellum	R	114	24	−50	−64
AD and Controls	Temporal pole, Inferior temporal gyrus, Middle temporal gyrus, Fusiform cortex	L	617	−46	2	−44
	Precuneus, Posterior cingulate cortex, Supracalcarine cortex	L & R	235	−14	−56	34
	Fusiform cortex, Inferior temporal gyrus, Middle temporal gyrus, Temporal pole	R	109	32	−8	−48
Regions that covary with Android: Gynoid Ratio						
bvFTD and Controls	Frontal pole, Superior frontal gyrus, Paracingulate gyrus, Anterior cingulate cortex	L & R	2465	10	56	24
	Temporal pole, Middle temporal gyrus, Orbitofrontal cortex, Insula, Frontal opercular cortex, Inferior frontal gyrus, Putamen	R	1764	48	10	−36
	Inferior frontal gyrus, Frontal opercular cortex, Insula, Central opercular cortex	L	704	−42	12	12
	Precuneus, Cuneus, Supracalcarine cortex, Lateral occipital cortex	L	656	−16	−72	24
	Central opercular cortex, Heschl’s gyrus, Parietal operculum, Insula, Postcentral gyrus, Supramarginal gyrus	R	567	52	−18	12
	Frontal pole, Medial prefrontal cortex	R	562	20	52	−10
	Cerebellum	L	384	−38	−70	−58
	Superior frontal gyrus, Precentral gyrus, Supplementary motor cortex, Middle frontal gyrus	R	347	18	−6	54
	Superior frontal gyrus, Middle frontal gyrus	R	296	22	18	58
	Temporal pole, Orbitofrontal cortex	L	274	−42	12	−42
	Caudate, Thalamus	R	212	24	−30	18
	Angular gyrus, Supramarginal gyrus, Lateral occipital cortex	R	196	66	−46	18
	Supramarginal gyrus, Postcentral gyrus	L	184	−66	−24	18
	Orbitofrontal cortex	L	169	−14	28	−24
	Cerebellum	L	168	−50	−50	−54
	Middle frontal gyrus, Precentral gyrus, Inferior frontal gyrus	R	165	42	12	34
	Cerebellum	R	145	20	−72	−50
	Precuneus, Cuneus, Supracalcarine cortex	R	104	20	−60	32
	Cerebellum	L	101	−14	−76	−30
AD and Controls	Frontal pole, Medial prefrontal cortex,	L & R	314	10	56	−24
	Lateral occipital cortex	R	151	36	−80	44
	Middle temporal gyrus, Inferior temporal gyrus	L	143	−62	−10	−32
	Temporal pole, Orbitofrontal cortex	R	121	18	12	−32
	Inferior frontal gyrus, Frontal opercular cortex	L	115	−56	30	8

All clusters reported using voxel‐wise contrasts and corrected for False Discovery Rate (FDR) at *P* < 0.05. All clusters reported at *t* > 1.79 with a cluster threshold of 100 contiguous voxels. B, Bilateral; BvFTD, behavioral‐variant frontotemporal dementia; L, Left; R, Right; MNI, Montreal Neurological Institute; SD, semantic dementia. Age is included as a nuisance variable in the analyses.

In AD, increasing BMI correlated with decreased grey matter volume in the left postcentral and precentral gyrus and central opercular cortex. VAT and android: gynoid ratio correlated with a more limited network involving the frontal pole, prefrontal cortex, temporal pole, and precuneus, predominantly left lateralized.

## Discussion

This study demonstrated that bvFTD is characterized by changes in body composition and fat deposition, indicating that this neurodegenerative disease affects systemic physiological functioning, beyond cognition and behavior. These changes in body composition were associated with atrophy involving a complex neural network, including structures known to be involved in reward processing and autonomic function. Despite being matched for BMI to controls, bvFTD participants exhibited increased fat deposition particularly in the android (trunk) area, shown by an increased android: gynoid ratio, and visceral adipose tissue area (VAT area). Increased fat deposition was correlated with increasing abnormal eating behavior and also overall behavioral change. Increasing visceral adipose tissue deposition and total fat mass also correlated with lower overall functional ability as measured by the FRS.

Undoubtedly, the abnormal eating behavior previously shown in FTD 2, 3, 4 plays a role in these changes in body composition, with increased android: gynoid ratio and VAT area correlating to abnormal eating behavior as measured by the CBI eating subscore. To put these changes down to abnormal eating behavior only may be overly simplistic. Indeed, increased android: gynoid ratio in controls has been found to be related to an increased incidence of the metabolic syndrome,^9^ which is characterized by insulin resistance and increased lipid levels. Increased regional fat deposition is more prone to undergo lipolysis and release lipids into the blood stream.^9^ An increased incidence of insulin resistance is found along the ALS‐FTD spectrum,^4^ with increased triglyceride levels and a higher cholesterol level related to an improved survival.^4^ In ALS, the metabolic syndrome is paradoxical as patients are well known to be hypermetabolic and experience increased energy expenditure,^4^ which has also been shown in FTD.^5^ One potential hypothesis for increased lipid levels in a state of hypermetabolism is that patients preferentially switch to peripheral lipid metabolism. This preferential switch to lipid as a source of energy has been shown in ALS animal models.^22^ It remains to be determined if the changes in body composition in FTD, as shown on DEXA scans, are a marker of changes in energy metabolism and lipid metabolism potentially providing a site for increased lipolysis and hence increased blood lipid levels,^9^ and/or contribute actively to modifying the neurodegenerative process. It is possible that these changes actively contribute to the neurodegenerative process with change in fat deposition in ALS found to improve survival.^7^

Another potential cause of changes in body composition in bvFTD may relate to autonomic dysfunction. The presence of autonomic dysfunction in FTD is increasingly recognized with reported changes in thermoregulation, pain sensation, and other symptoms related to autonomic dysfunction.^23^, ^24^ Changes in heart rate variability have also been related to abnormalities in key central structures known to control autonomic function including the insula and anterior cingulate cortex.^5^

The current study provides evidence that autonomic dysfunction may play a role in changes in body composition, potentially through distributed neural networks. Here, increasing VAT area and android: gynoid ratio in bvFTD correlated with decreasing grey matter volume in a distributed network central to autonomic function including the temporal pole, hippocampus, amygdala, insula and anterior cingulate cortex, parietal regions, and precuneus.^25^ These structures, together with the nucleus accumbens, are known to be integral to autonomic control of the human body and known to undergo pathological changes in FTD. The evidence in FTD for potential autonomic involvement affecting body composition adds further evidence to that arising from studies in healthy individuals, which showed that autonomic control influences the deposition of adipocytes and body weight regulation.^26^, ^27^ Further studies are required in FTD where changes in autonomic function are examined alongside measures of body composition to confirm this potential correlation.

Neural network changes were not only limited to structures involved in autonomic control, but also extended to structures involved in reward evaluation including the insula, caudate, putamen, nucleus accumbens, and thalamus.^28^, ^29^, ^30^ The thalamus plays a crucial role in goal‐directed behavior and reward processing as part of a wider network involving the ventromedial prefrontal cortex, ventral striatum, amygdala, and anterior insula.^31^, ^32^ The thalamus is consistently implicated in a variety of reward‐based behavioral changes in FTD including eating behavior^3^ and sexual behaviour.^33^ The thalamus exhibits dense connections to the hypothalamus^34^ which may also potentially influence changes in body composition through neuroendocrine inputs, and has been previously implicated in changes in eating behavior in FTD and BMI in ALS.^2^, ^35^ Changes in body composition (android: gynoid ratio and VAT area) also correlated to atrophy of the cerebellum, which has again been implicated in overeating behavior in FTD,^3^ and a range of autonomic, cognitive, emotional functions,^36^, ^37^, ^38^ and reward processing.^39^

The distributed neural networks relevant to body composition identified here align with previous work indicating that metabolic and eating changes in FTD involve an interaction between autonomic and reward processing. We have previously shown that increased food intake in FTD during a test meal correlated with changes in the cingulate cortices, thalami, and cerebellum, structures controlling cognitive‐reward, autonomic, neuroendocrine, and visual modulation of eating behavior.^3^ Further studies are needed to ascertain the interactions between reward processing and autonomic function on body composition and whether these changes are driven by changed eating behavior, or are part of the neurodegenerative process.

The neural correlate changes in BMI were less extensive than those shown for body composition with changes limited to structures involved in autonomic control including the anterior cingulate cortex, temporal pole, insula, and parietal regions in bvFTD and in the pre‐ and postcentral gyrus and central opercular cortex in AD, which have been proposed to be connected to the insula.^40^ These findings suggest that BMI provides only a snapshot of the central changes occurring in neurodegeneration, while body composition changes provide a far more comprehensive insight into the central physiological changes potentially occurring between reward and autonomic control.

Further studies are required to ascertain when changes in body composition occur in FTD, their progression over time, and effect on overall survival. Indeed, we need to ascertain whether these changes are the result of increased caloric intake or related to the intake of certain nutrients (e.g., fat), and the potential neuroendocrine effects related to changes in body composition (e.g., leptin levels). Studies are required in both human and animal models to examine the interactions between intake, metabolic rate, neuroendocrine changes, and effect on underlying pathology and disease progression. Whether neuroendocrine changes are also related to sex particularly involving the hypothalamus and its interactions between the pituitary and gynoid axis, and how these could modulate changes in body composition will also deserve attention. Studies will also need to consider the interactions between environmental factors (such as diet), genetic factors, and metabolic changes, and the effect that these factors have on underlying cell processing including oxidative stress and inflammation, and their resulting effects on the neurodegenerative process.^41^, ^42^

The current study has demonstrated that the clinical phenomenology in bvFTD is not limited to cognitive and behavioral changes. Our findings demonstrate the presence of systemic changes including changes in body composition and fat deposition, that potentially provide insights into the interaction between autonomic and reward processing that are emerging as core deficits in FTD. Changes in body composition may provide potential markers of underlying network dysfunction, that could be harnessed to monitor disease progression and used in clinical trials to monitor and modify, in order to slow the course of these devastating conditions.

## Author Contributions

Rebekah Ahmed: study concept, data analyses, manuscript preparation, and writing. Ramon Landin‐Romero: data analyses, manuscript preparation, and writing. Cheng Liang: data analyses, manuscript preparation, and writing. Julia Keogh: data analyses, manuscript preparation, and writing. Elana Henning: data analyses, manuscript preparation, and writing. Cherie Strikwerda‐Brown: data analyses, manuscript preparation, and writing. Emma Devenney: data analyses, manuscript preparation, and writing. Matthew C Kiernan: data analyses, manuscript preparation, and writing. John Hodges: data analyses, manuscript preparation, and writing. Sadaf Farooqi: study concept, data analyses, manuscript preparation, and writing. Olivier Piguet: data analyses, manuscript preparation, and writing.

## Conflict Of Interest

The authors declare no competing financial interests. Rebekah Ahmed, Ramon Landin‐Romero, Cheng Liang, Julia Keogh, Elana Henning, Cherie Strikwerda‐Brown, Emma Devenney, Matthew C Kiernan, John Hodges, I. Sadaf Farooqi and Olivier Piguet have no disclosures.

## Supporting information


**Figure S1.** Patterns of atrophy within and between patient groups. Group results from voxel‐based morphometry analyses illustrating areas of greater decreased grey matter density in (A) bvFTD patients compared to controls in blue, and (B) AD patients compared to controls in red. (C) Comparisons between patient groups illustrate greater reduction in grey matter intensity in bvFTD patients in blue, and in AD patients in red. All analyses are reported at *P* < 0.005 voxel‐wise, uncorrected for multiple comparisons with minimum cluster size of 100 voxels. The left side of the image is the left side of the brain. Numbers below each slice refer to MNI z‐coordinates.

**Table S1**. Behavioral‐variant FTD patients showed widespread atrophy in frontal and anterior‐temporal regions compared to controls, while AD patients showed widespread atrophy in temporal and posterior regions compared to controls. Comparisons between patient groups revealed reduced grey matter density in bvFTD in bilateral orbitofrontal cortex, frontal pole, and cerebellum, while AD showed reduced grey matter density in bilateral posterior cingulate cortex and temporo‐parietal junction.
