# Supplementary material for: Neural networks associated with body composition in frontotemporal dementia
Source: Ann Clin Transl Neurol. 2019 Aug 28;6(9):1707–17. doi: 10.1002/acn3.50869 (PMC6764740; doi:10.1002/acn3.50869)
Supplement: Supplementary file 1 — Figure S1. Patterns of atrophy within and between patient groups. Group results from voxel‐based morphometry analyses illustrating areas of greater decreased grey matter density in (A) bvFTD patients compared to controls in blue, and (B) AD patients compared to controls in red. (C) Comparisons between patient groups illustrate greater reduction in grey matter intensity in bvFTD patients in blue, and in AD patients in red. All analyses are reported at P < 0.005 voxel‐wise, uncorrected for multiple comparisons with minimum cluster size of 100 voxels. The left side of the image is the left side of the brain. Numbers below each slice refer to MNI z‐coordinates. [file ACN3-6-1707-s001.docx]

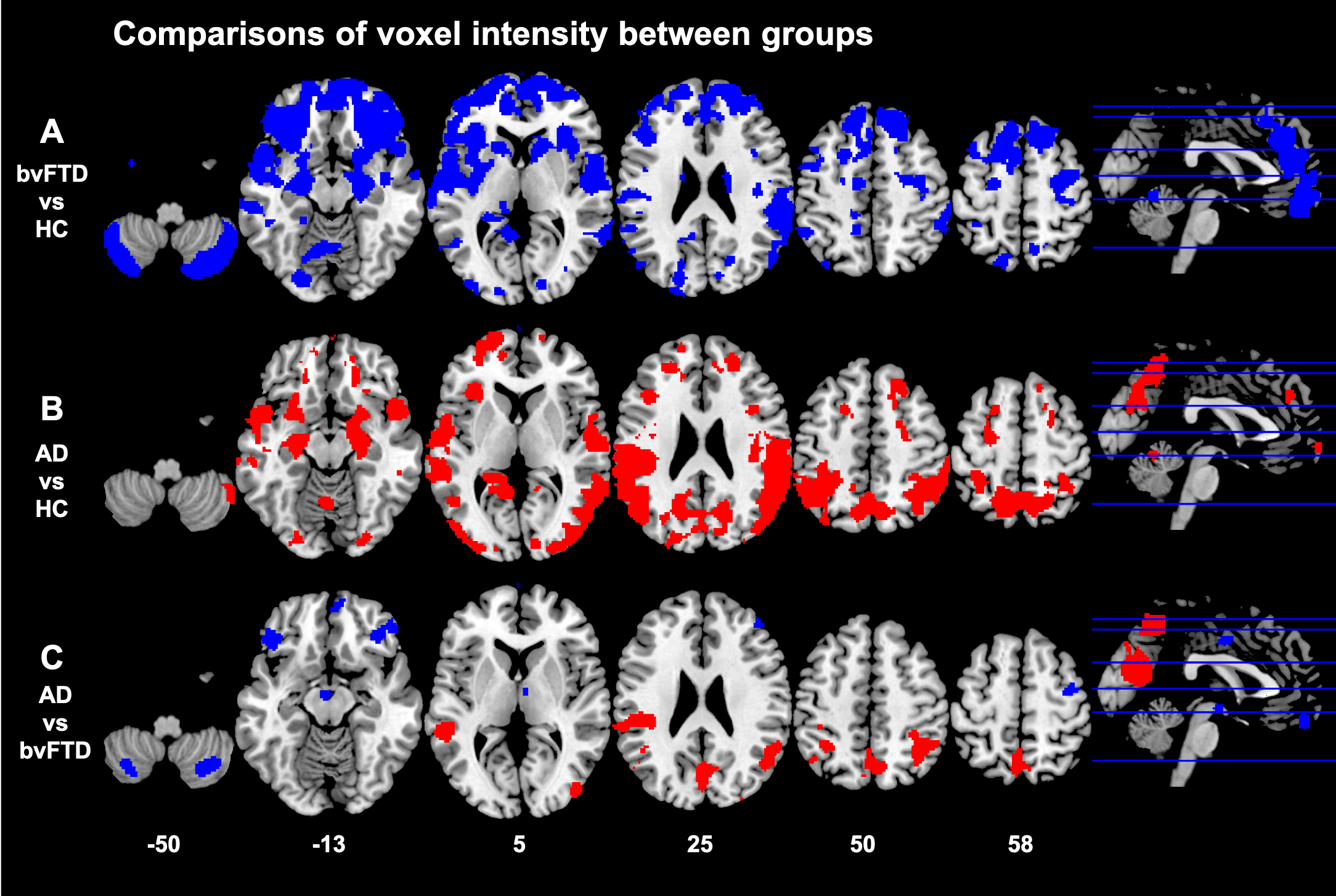


**Supplementary Figure 1. Patterns of atrophy within and between patient groups.** Group results from voxel-based morphometry analyses illustrating areas of greater decreased grey matter density in **(A)** bvFTD patients compared to controls in blue, and **(B)** AD patients compared to controls in red. **(C)** Comparisons between patient groups illustrates greater reduction in grey matter intensity in bvFTD patients in blue, and in AD patients in red. All analyses are reported at *p* < .005 voxel-wise, uncorrected for multiple comparisons with minimum cluster size of 100 voxels. The left side of the image is the left side of the brain. Numbers below each slice refers to MNI z-coordinates.
