# Supplementary material for: Neural networks associated with body composition in frontotemporal dementia
Source: Ann Clin Transl Neurol. 2019 Aug 28;6(9):1707–17. doi: 10.1002/acn3.50869 (PMC6764740; doi:10.1002/acn3.50869)
Supplement: Supplementary file 2 — Table S1. Behavioral‐variant FTD patients showed widespread atrophy in frontal and anterior‐temporal regions compared to controls, while AD patients showed widespread atrophy in temporal and posterior regions compared to controls. Comparisons between patient groups revealed reduced grey matter density in bvFTD in bilateral orbitofrontal cortex, frontal pole, and cerebellum, while AD showed reduced grey matter density in bilateral posterior cingulate cortex and temporo‐parietal junction. [file ACN3-6-1707-s002.docx]

Behavioural Variant FTD patients showed widespread atrophy in frontal and anterior-temporal regions compared to controls, while AD patients showed widespread atrophy in temporal and posterior regions compared to controls. Comparisons between patient groups revealed reduced grey matter density in bvFTD in bilateral orbitofrontal cortex, frontal pole and cerebellum, while AD showed reduced grey matter density in bilateral posterior cingulate cortex and temporo-parietal junction.

|  | | | | **MNI coordinates** | | |
| --- | --- | --- | --- | --- | --- | --- |
|  | **Regions** | **Side** | **Number of voxels** | **x** | **y** | **z** |
| **AD > bvFTD** |  |  |  |  |  |  |
|  | Cerebellum - VIIIa & VIIb | R | 278 | 28 | -68 | -58 |
|  | Frontal pole, frontal medial cortex | R | 228 | 0 | 56 | -26 |
|  | Frontal orbital cortex, frontal pole | R | 191 | 42 | 32 | -18 |
|  | Frontal orbital cortex, frontal pole | L | 184 | -34 | 30 | -20 |
|  | Frontal pole, middle frontal gyrus | R | 163 | 44 | 44 | 16 |
|  | Cerebellum - VIIb | L | 157 | -26 | -72 | -56 |
|  | R thalamus, L brainstem, L midbrain | L&R | 119 | -4 | -12 | -14 |
|  | Anterior cingulate cortex | R | 119 | 2 | 8 | 30 |
|  | Precentral gyrus, middle frontal gyrus | R | 112 | 38 | -10 | 54 |
| **bvFTD > AD** |  |  |  |  |  |  |
|  | Intracalcarine cortex, supracalcarine cortex, cuneal cortex, precuneous cortex, lingual cortex | L&R | 1244 | 4 | -76 | 8 |
|  | Inferior and superior lateral occipital cortex, angular gyrus, supramarginal gyrus and middle temporal gyrus | R | 1047 | 50 | -80 | -6 |
|  | Angular gyrus, superior parietal lobule, superior lateral occipital cortex | R | 622 | 54 | -52 | 34 |
|  | Lateral occipital cortex, angular gyrus, posterior supramarginal gyrus, postcentral gyrus, superior parietal lobule | L | 580 | -52 | -66 | 24 |
|  | Planum temporale, parietal operculum cortex, anterior supramarginal gyrus | L | 492 | -48 | -32 | 10 |
|  | Posterior superior temporal gyrus, middle temporal gyrus, supramarginal gyrus | L | 229 | -50 | -42 | -2 |
| **Con > AD** |  |  |  |  |  |  |
|  | Anterior temporal fusiform cortex, temporal pole, frontal orbital cortex, Herschl's gyrus, insular cortex, precentral gyrus, planum temporale, parietal operculum cortex, anterior and posterior supramarginal gyrus, middle temporal gyrus, angular gyrus, superior parietal lobule, anterior and posterior superior temporal gyrus, planum polare, postcentral gyrus, angular gyrus, superior and inferior lateral occipital cortex | L&R | 29895 | 34 | -2 | -40 |
|  | Anterior parahippocampal gyrus, hippocampus, amygdala, frontal orbital cortex | R | 829 | 20 | -16 | -28 |
|  | Cerebellum - crus I, V, VI | L | 767 | -52 | -58 | -46 |
|  | Superior frontal gyrus, precentral gyrus, middle frontal gyrus | L | 491 | -20 | 12 | 46 |
|  | Anterior parahippocampal gyrus, hippocampus, amygdala | L | 446 | -18 | -18 | -28 |
|  | Precentral gyrus, pars opercularis | L | 166 | -50 | 6 | 14 |
|  | Cerebellum - VIIIa, VIIb, Crus I, Crus II | R | 160 | 42 | -52 | -58 |
|  | Thalamus, caudate | L | 131 | -22 | -28 | 12 |
| **Con > bvFTD** |  |  |  |  |  |  |
|  | Frontal pole, frontal medial cortex, anterior cingulate cortex, frontal orbital cortex, paracingulate gyrus, superior frontal gyrus, middle frontal gyrus, inferior frontal gyrus, insular cortex, temporal pole, insular cortex, caudate, hippocampus, amygdala, putamen, frontal operculum cortex, central opercular cortex, planum polare, anterior and posterior temporal fusiform cortex, anterior parahippocampal gyrus, Hescl's gyrus, anterior superior temporal gyrus, middle temporal gyrus, supramarginal gyrus, angular gyrus, parietal operculum cortex, lateral occipital cortex, | L&R | 40669 | -30 | 2 | -52 |
|  | Cerebellum - VIIb, crus I, crus II, V, VI, Lingual gyrus, posterior cingulate gyrus, hippocampus, parahippoampal gyrus | L | 3167 | -28 | -72 | -60 |
|  | Cerebellum - VIIIa, VIIb, VI, crus I, crus II | R | 1881 | 22 | -60 | -64 |
|  | Occipital fusiform gyrus, occipital pole | L | 1021 | -22 | -90 | -16 |
|  | Precentral gyrus, superior frontal gyrus | L | 454 | -24 | -8 | 46 |
|  | Lateral occipital cortex, superir parietal loble | L | 429 | -14 | -72 | 52 |
|  | Precuneous cortex | L | 408 | -8 | -52 | 18 |
|  | Precuneous cortex, lateral occipital cortex, superior parietal lobule | R | 229 | 8 | -66 | 54 |
|  | Cuneal cortex, precuneous cortex | R | 223 | 12 | -80 | 20 |
|  | Superior parietal lobule, angular gyrus | L | 182 | -38 | -52 | 40 |
|  | Occipital fusiform gyrus, occipital pole, intacalcarine sulcus, lingual gyrus | R | 174 | 18 | -88 | -8 |
|  | Precuneous cortex, posterior cingulate cortex | R | 110 | 12 | -54 | 30 |

> denotes group that has greater grey matter intensity
